# Impact of Flavonoids on Cellular and Molecular Mechanisms Underlying Age-Related Cognitive Decline and Neurodegeneration

**DOI:** 10.1007/s13668-018-0226-1

**Published:** 2018-04-27

**Authors:** Emma Flanagan, Michael Müller, Michael Hornberger, David Vauzour

**Affiliations:** Norwich Medical School, Faculty of Medicine and Health Sciences, University of East Anglia, Norwich, NR4 7UQ UK

**Keywords:** Polyphenols, Memory, Alzheimer’s disease, Signalling pathways, Microbiome

## Abstract

**Purpose of Review:**

This review summarises the most recent evidence regarding the effects of dietary flavonoids on age-related cognitive decline and neurodegenerative diseases.

**Recent Findings:**

Recent evidence indicates that plant-derived flavonoids may exert powerful actions on mammalian cognition and protect against the development of age-related cognitive decline and pathological neurodegeneration. The neuroprotective effects of flavonoids have been suggested to be due to interactions with the cellular and molecular architecture of brain regions responsible for memory.

**Summary:**

Mechanisms for the beneficial effects of flavonoids on age-related cognitive decline and dementia are discussed, including modulating signalling pathways critical in controlling synaptic plasticity, reducing neuroinflammation, promoting vascular effects capable of stimulating new nerve cell growth in the hippocampus, bidirectional interactions with gut microbiota and attenuating the extracellular accumulation of pathological proteins. These processes are known to be important in maintaining optimal neuronal function and preventing age-related cognitive decline and neurodegeneration.

## Introduction

Advances in medical science over the last century have resulted in a considerable increase in human life expectancy. Despite this positive outcome, with increasing age comes an increased susceptibility to chronic organ disease and decline of metabolic and immune functions with impact on the brain [1]. Although some decline in cognitive function does occur with normal ageing, there is also an increased age-associated risk neurodegenerative disorders of which Alzheimer’s disease (AD) is the most prevalent. As such, with the global ageing population, the prevalence of dementia worldwide is estimated to double every 20 years, and expected to increase to 115 million affected individuals by 2050 [2]. Existing drug treatments for neurodegenerative conditions rarely curtail the underlying disease processes, and consequently, there is an urgent need to develop alternative strategies to directly prevent, slow and even stop neurodegeneration. Lifestyle strategies such as nutritional interventions have potential to be a safe, cheap and effective alternative to protect against age-related cognitive decline and neurodegeneration, resulting in significant personal and societal benefits [3]. In particular, there has been a growing recent focus on the potential for dietary flavonoids, plant-derived compounds found abundantly in fruit, vegetables, cocoa and certain beverages such as coffee and tea, to directly prevent pathological mechanisms underlying neurodegeneration [4, 5]. This review aims to summarise the existing evidence in favour of dietary flavonoids as a viable alternative approach to directly impact cognitive decline and neurodegenerative disease, with a particular focus on AD.

## Flavonoids, Age-Related Cognitive Decline and Neurodegeneration

Even in the absence of pathological neurodegeneration, age-related cognitive decline has been consistently demonstrated in studies involving both animals and humans. Alterations in cognition appear to occur predominantly in domains relating to storage of newly acquired information including short-term memory [6], working memory and executive function [7]. The overall reduced risk of cognitive decline that has been observed with higher intakes of fruit and vegetables is likely attributable to a higher intake of specific flavonoids [8]. In line with this, in middle-aged adults a higher total intake of flavonoids has been associated with better episodic memory and language performance [9], and greater cognitive performance at baseline with less decline at follow-up in non-demented older adults [10]. These findings have encouraged further investigation into specific flavonoids, their possible health benefits and the mechanisms by which they might exert these effects.

Berries have received particular attention for preventing age-related cognitive decline due to their considerably high concentrations of flavonoids. Blueberries are particularly rich in flavonoids, the most prevalent being anthocyanidins (delphinidin, cyanidin, petunidin, peonidin and malvidin), flavanols (catechin, epicatechin, procyanidins B type) and flavonols (quercetin and myricetin) [11]. In aged rodents, blueberry supplementation has been shown to improve spatial memory [12], object recognition memory [13] and inhibitory fear conditioning learning [14]. Blueberry appears to have a pronounced effect on short-term memory [15] and has also been shown to improve long-term reference memory following 8 weeks of supplementation in aged rats [12, 16]. Regarding studies involving humans, a long-term prospective study of neurologically healthy older adults observed a greater intake of berry anthocyanidins related to a slower rate of cognitive decline and was associated with a 2.5-year delay in the onset of deficits [17]. In addition to berries, animal studies involving flavanols from cocoa and tea provide positive evidence that these flavonoids could also protect against cognitive decline. For example, (−)-epicatechin enhanced the retention of spatial memory in male C57BL/6 mice (8–10 weeks old), particularly in combination with exercise [18], and a 6-month administration of green tea catechins in 14-month-old female C57BL/6 mice prevented spatial learning and memory decline [19].

Regarding pathological cognitive decline, approximately 60 to 80% of dementia cases are due to AD [20], affecting over than 25 million people globally [21]. AD is diagnosed based on a combination of clinical history, often provided by a close family member, and performance on neuropsychological testing with a particular focus on short-term memory performance. Mild cognitive impairment (MCI) is characterised by cognitive impairment in the absence of significant disruption to everyday function [22], and individuals diagnosed with MCI have an increased risk for progressing to AD [23, 24]. Several epidemiological studies have investigated the associations between higher intakes of dietary flavonoids and possible neuroprotective effects in the context of dementia. For example, higher intake of flavonoid-rich foods was associated with a significantly lower risk of dementia in a large cohort of older adults aged 65 years and over [25].

Gene mutations in encoding the amyloid precursor protein (APP) or presenilin (PS1 and PS2) [26], and more recently the *APOE* gene with the *E4* allele mutation, have been identified as significant genetic susceptibility predictors for AD [27], with over 60% of individuals affected by AD carrying the *APOE4* variant compared to a much lower prevalence of 25–27% in the general population. The *APOE4* genotype is associated with more rapid cognitive decline [28] and poorer performance even in neurologically healthy carriers [29]. The *APOE4* genotype is also associated with an earlier age of disease onset [28] and a higher conversion rate to AD in individuals diagnosed with MCI [30]. Of particular relevance is emerging evidence suggesting that the *APOE4* genotype may modify the beneficial effect of flavonoids on cognition and prevalence of AD. For example, a greater consumption of flavonoid-rich fruits and vegetables was associated with a decreased risk of dementia especially amongst APOE4 non-carriers in the Three-City cohort study [8]. By contrast, in the Kame project, a more frequent consumption of flavonoids-rich foods was suggested to delay the onset of AD particularly in *APOE4* carriers [31]. As such, further investigation is required in order to elucidate the exact mechanisms by which the presence of the *APOE4* mutation modulates the health benefits from dietary flavonoids.

Results from intervention studies involving humans are less prevalent. Previous acute human studies have shown that cocoa flavanol consumption improved working memory and attention [32, 33], significantly increased cerebral blood flow across the brain [34], particularly in the dentate gyrus [35], in healthy adults. A randomised, placebo-controlled trial of Concord grape juice in older adults with memory decline showed improvement on verbal encoding [36], and similar effects were observed with blueberry juice administered over 12 weeks [37]. Similarly, Desideri et al. (2012) also observed better verbal fluency performance in older individuals with MCI following consumption of a drink high in flavan-3-ol over only 8 weeks [38]. As such, the consumption of flavonoid-rich foods may have the potential to limit or even reverse age-related cognitive decline. However, findings that lend support to the causal effects of dietary flavonoids on the prevention of age-related cognitive decline and dementia from interventional studies still remains sparse. Given the promising results from epidemiological and preclinical studies, further attention needs to be dedicated to determining the causality of flavonoid consumption on improving cognition and preventing dementia.

## Mechanisms Underlying the Neuroprotective Effects of Flavonoids

Despite the precise causes of cognitive decline remaining unclear, age-related neurodegeneration is considered to be underpinned by several interlinked cellular and molecular mechanisms, including cumulative damage due to chronic neuroinflammation, oxidative stress, impaired mitochondrial function, activation of neuronal apoptosis, deposition of aggregated proteins and excitotoxicity. The health-promoting effects of flavonoids have previously been attributed to their ability to reduce cell damage by directly scavenging free radical species, according to evidence from in vitro studies. However, the concentrations of flavonoids that have been found to exert such antioxidant activity are significantly higher than can be achieved through diet in humans. Furthermore, many flavonoids have very limited bioavailability as they are efficiently metabolised before being able to exert their antioxidant effects [39]. Recent evidence suggests that flavonoids at physiologically attainable concentrations may exert different activities which directly impact neurodegenerative disease-causing processes. The possible mechanisms by which flavonoids prevent against age-related neurodegeneration are explored in further detail here.

### Reduction of Neuropathological Protein Accumulation

The neuropathological hallmarks of AD are the extracellular deposition of amyloid plaques and the intracellular accumulation of hyperphosphorylated tau proteins—a neuronal microtubule-associated protein regulated by phosphorylation of various protein kinases [40]. Most preclinical studies investigating the effects of flavonoids have focused on models where there is increased production of beta-amyloid (Aβ), a small protein produced by the enzymatic cleavage of APP [41]. One way in which flavonoids may prevent the accumulation of Aβ pathology is by preventing neuronal apoptosis triggered by neurotoxic processes through the inhibition of β-secretase (BACE-1) [42] and activation of α-secretase (ADAM10) [43]. However, these in vitro studies involved flavonoid concentrations far greater than those encountered in vivo following a normal diet. In animal studies, epigallocatechin-3-gallate (EGCG) administered for 6 months significantly reduced cognitive decline and Aβ in an AD mouse model, [44] and green tea catechin administration improved spatial learning and memory in senescence-prone mice [45], the latter being associated with decreased in Aβ accumulation and upregulation of proteins related to synaptic plasticity in the hippocampus. In addition, administration of a catechin-rich grape seed extract was associated with reduced cognitive decline in conjunction with decreased concentrations of Aβ oligomers [46]. Similarly, administration of the citrus-derived flavone nobiletin in APP-SL 7-5 Tg mice was associated with reduced hippocampal accumulation of neurotoxic Aβ proteins [47]. Mechanisms by which some flavonoids may prevent Aβ plaque accumulation include inhibition of amyloid aggregation and fibrillization [46], either due to metal chelation activity [48] or through facilitating production of non-toxic oligomers [49], as well as the upregulation of α-secretase through modulation of A disintegrin and metalloproteinaise domain-containing protein 10 (ADAM10) [50].

Despite these promising findings, it is important to note that the presence of Aβ pathology and cognitive deficits are not always well correlated. For example, a recent study observed cognitive improvement following a polyphenol-rich diet without associated changes in classical AD neuropathology [51••]. It is possible that some of the protective effects of flavonoids on age-related cognitive decline could instead be, at least in part, due to downstream processes from changes in Aβ, such as tau phosphorylation and fibrillization. The development of neurofibrillary tangles could be inhibited by flavonoids such as (-)-epicatechin and hesperetin, through their promotion of protein kinase B, or Akt, phosphorylation leading to reduced GSK3β-driven hyperphosphorylation of tau [52, 53]. As such, the benefits of dietary flavonoids in the context of AD may extend beyond interactions with processes underpinning neurotoxic Aβ accumulation.

### Stimulation of Neuronal Signalling Pathways and Synaptic Plasticity

Although the precise sites of the interactions between flavonoids and neuronal signalling pathways remain to be determined, based on existing evidence flavonoids may exert their effects through (1) modulating signalling cascades that control neuronal apoptosis; (2) modulating the expression of specific genes and (3) impacting mitochondrial activity [54]. In particular, current findings suggest that flavonoids impact on the extracellular signal-regulated kinase (ERK) pathway [52], which appears to be mediated by interactions with mitogen-activated protein kinase (MEK) 1 and 2, and potentially membrane receptors [55]. Evidence from in vitro studies has suggested that flavonoids increase the activation of ERK. For example, both the flavanol (−)-epicatechin [56] and the citrus flavanone hesperetin [57] were observed to activate ERK1/2 in cortical neurons at nanomolar concentrations, and submicromolar concentrations of EGCC were reported to restore ERK1/2 activities in 6-hydroxydopamine treated or serum-deprived neurons [58]. ERK activation often subsequently leads to the activation of the CREB transcription factor, considered critical in supporting synaptic plasticity [59] and controlling neuronal survival by regulating the expression of a number of important genes, including brain-derived neurotrophic factor (BDNF) [60]. Flavonoids are also known to modulate the activity of the Akt enzyme system (also known as PKB), regulated by phosphoinositide 3-kinase (PI3K). Hesperitin, a flavonone from citrus fruits, has been observed to activate Akt/PKB and subsequently inhibits activation of proteins associated with neuronal apoptosis including apoptosis signal-regulating kinase 1 (ASK1), Bad, caspase-9 and caspase-3 [57]. Furthermore, flavonoid-elicited activation of Akt in hippocampal neurons has been observed to result in increased mRNA translation of the activity-regulated cytoskeletal-associated protein (Arc/Arg3.1) [15]. Increased Arc expression may facilitate changes in synaptic strength and morphology of dendritic spines [61], and indeed, in vitro studies have indicated that changes in neuronal morphology and dendrite growth occur in response to flavonoid supplementation [62].

### Neuroinflammation

A normal inflammatory response is critical for supporting health and in particular the brain’s defence against damage. However, a chronic upregulation of neuroinflammation, indicated by increased circulatory pro-inflammatory cytokines and biomarkers, may contribute to a cascade of events resulting in progressive neuronal damage [63]. Chronic neuroinflammation can interfere with proper neuronal functioning, impede episodic memory encoding and facilitate pathological accumulation and impact of Aβ plaques. Chronically elevated activation of pro-inflammatory cytokines, such as tumour necrosis factor (TNF-α), contributes to neuronal injury through amplification of the inflammatory response [64]. Indeed, individuals with MCI have been found to have elevated circulating levels of serum TNF-α compared to age-matched controls [65], and is over expressed in affected neural regions [66] and in cerebrospinal fluid (CSF) of individuals diagnosed with AD [67]. Furthermore, the acute phase protein, serum C-reactive protein (CRP), is associated with greater risk of dementia onset [68] and memory impairment, and has also been found to co-localise with pathological Aβ and neurofibrillary tangles in the brains of AD patients [69, 70]. Indeed, elevated plasma concentrations of CRP have been consistently found in individuals with MCI [71] and AD [72].

Neuroinflammation has further been implicated in contributing to AD pathology through increased activation of microglial and consequently elevated activation of acetyl cholinesterase (AChE) and free radical generation [73]. Indeed, findings of lower AD risk associated with long-term use of non-steroidal anti-inflammatory drugs (NSAIDs) [74] have led to increased attention turning to developing anti-inflammatory pharmaceutical solutions to reduce the impact of neuroinflammation on brain disease. Flavonoids may also prevent neuroinflammation via several anti-inflammatory mechanisms, including (1) inhibiting the microglial activation of inflammatory cytokines, including TNF-α and IL-1β; (2) inhibiting iNOS induction and subsequent nitric oxide production in response to glial activation; (3) inhibiting activation of NADPH oxidase and subsequent ROS generation in activated glia; and (4) downregulating activity of pro-inflammatory transcription factors such as NF-κB through modulation of glial and neuronal signalling pathways [75]. However, the majority of evidence from in vitro research has come from studies using single flavonoids, typically aglycones, at supraphysiological concentrations. Few studies however have investigated the anti-inflammatory effects of physiologically attainable flavonoid concentration in healthy subjects, and the findings from these are less consistent [76]. However, epicatechin and catechin were observed to inhibit TNF-α release but not iNOS expression or nitric oxide production in primary glial cells [77], providing promising evidence that some flavanols at physiologically relevant concentrations could exert anti-inflammatory effects. In studies involving humans, higher intake of a flavonoid-rich was associated to lower levels of inflammatory biomarkers including CRP, IL-6 and adhesion factors [78]. Furthermore, total flavonoid intake as well as intake of the specific flavonols anthocyanidins and isoflavones were related to lower blood levels of CRP in a large cross-sectional epidemiological study [79]. Finally, blueberry anthocyanin given to adults aged 40–74 years over 3 weeks significantly reduced plasma concentrations of NF-kB-related pro-inflammatory cytokines and chemokines (IL-4, IL-13, IL-8 and IFN-α) [80]. In contrast, a short-term intervention with black tea (900 mL/day, 4 weeks) showed no improvements in plasma CRP concentrations in patients with coronary artery disease [81]. Furthermore, no significant effect was observed in plasma CRP or ICAM-1 levels in healthy adults consuming diets rich in berries and apples for 6 weeks [82]. The inconsistent results from these various trials on the preventive anti-inflammatory effects of flavonoid supplementation reinforce the necessity for more prospective randomised trials with larger sample sizes and under clinical conditions.

### Vascular Function and Angiogenesis

The integrity of the vascular system becomes increasingly vital with increasing age. The risk factors for reduced vascular health and function, such as diabetes mellitus, smoking, hypertension and arteriosclerosis, are shared by several forms of dementia including AD [83]. There have been findings that flavonoid-rich diets are associated with lower cardiovascular risk through lowering blood pressure, increasing the bioavailability of nitric oxide [84] and improving arterial flow-mediated dilation [85]. Vascular function within the brain is not only integral for the prevention of ischaemic events, but also for maintaining cerebral blood flow underpinning cognitive function. Indeed, evidence suggests that flavonoids can improve cerebrovascular blood flow through their impact on the peripheral and cerebral vascular system [86]. Neuroimaging studies in both healthy older [34, 35, 86, 87] and younger adults [88] have shown that the consumption of flavanol-rich cocoa could significantly enhance cerebral blood flow in response to cognitive tasks. Additionally, significantly increased perfusion in the middle and inferior right frontal gyrus was observed following the intake of a high-flavonoid citrus drink in healthy adults after 2 h compared to baseline and controls [89•]. These effects are particularly significant, as increased cerebrovascular function is known to facilitate adult neurogenesis [90], especially within the hippocampus, and to enhance vascularisation [91], two events important in the maintenance of cognitive performance. If such effects prove possible, then diet would have the potential not only to slow the progression of neurodegeneration and cognitive decline but also to potentially reverse cognitive impairment through stimulation of neuronal growth in the hippocampus.

### Interactions with the Microbiome

The gastrointestinal tract plays a key role in protecting and promoting health, including regulating energy metabolism, acting as a barrier to potential toxic compounds present in ingested food and supporting the immune system. The functioning of microbiota in particular can directly impact physiological processes throughout the body. Research over the past two decades has indicated that the gut microbiome and its interaction with dietary compounds has important implications for human health. Dietary flavonoids can have a direct effect on the gastrointestinal tract and particularly interact with the gut microbiota. Flavonoids reach the stomach and intestine in high concentrations, enabling them to exert their most direct effects here, including potential antioxidant effects, before being processed by the liver. Flavonoids that are not absorbed in the small intestine due to their close binding with β-glucosides [92] and other sugars are then broken down in the colon by microbiota into phenolic acids and other metabolites. Flavonoids and their metabolites are understood to regulate the function of the gastrointestinal tract through direct interactions with the gut microbiota. Most notably, flavonoids can impact the composition of bacterial populations in the gastrointestinal tract, including promoting the growth of beneficial (commensal) bacteria and potentially inhibiting pathogenic strains. The impact of flavonoids on specific strains of bacteria is considered to be dependent on the molecular structure of certain classes of flavonoid [93]. For example, A-type proanthocyanidins present in cranberries have been found to inhibit the adhesion of *Escherichia coli* bacterial strains within the human urinary tract [94]. Quercetin has also been found to inhibit the growth of *Ruminococcus gauvreauii*, *Bacteroides galacturonicus* and *Lactobacillus* sp. strains [95], and flavonoids present in berries have also shown inhibitory actions against *Bacillus cereus*, *Campylobacter jejuni*, *Clostridium perfingens*, *Helicobacter pylori*, *Staphylococcus aureus*, *Staphylococcus epidermidis* and *Candida albicans* [96]. Conversely, a diet of apples genetically modified to have higher concentrations of flavonoids was associated with greater concentrations of beneficial strains of *Bifidobacterium* spp. and Bacteroides-Prevotella-Poryphyromonas in mice [97].

The interactions between flavonoids and microbiota are bidirectional, and microbiota in the gastrointestinal tract can impact the absorption and bioavailability of flavonoids present in ingested food, which integrally modifies their impact on health. In addition to gastrointestinal enzymes, microbiota are also involved in the metabolism of flavonoids. Proanthocyanidins are poorly absorbed within the gastrointestinal tract, however, once they are metabolised by the microbiota into smaller compounds such as phenolic acids that are readily absorbed [98]. These flavonoid metabolites are then able to be absorbed into the bloodstream where they are able to exert more systemic health effects. For example, the gut metabolites of anthocyanins have been observed to attenuate the adhesion of monocytes to TNFα-activated endothelial cells, which suggests they could play a key role in preventing the development of atherosclerosis at its earliest stages [99•]. Flavonoid metabolites, particularly in certain combinations, have been suggested to be able to impact health differently to their parent flavonoid compounds [100–101, 102•]. In particular, specific combinations of flavonoid metabolites were found to significantly reduce IL-Iβ secretion, suggesting further potential anti-inflammatory effects of flavonoids their metabolism [102•]. Furthermore, evidence regarding phenolic acids metabolised from anthocyanidins by microbiota has suggested that they could interfere with the assembly of Aβ in rats [103••]. However, more work is required to more fully understand the complex interactions between flavonoids and the microbiome and to determine the exact nature of their subsequent local and systemic health benefits, particularly in humans.

## Conclusion

With an ageing population, age-related cognitive decline and neurodegeneration pose a significant challenge for the future. Dementia costs to the UK alone have been estimated to be £26 billion each year, with the cost expected to double in the next 25 years to £55 billion by 2040 [104]. A reduction in age-related cognitive decline by just 1% per year would cancel out all estimated increases in the long-term care costs due to our ageing population [104]. The potential neuroprotective benefits of dietary flavonoids could provide a promising alternative strategy to combat age-related cognitive decline and pathological neurodegeneration. However, given the lack of intervention trials conducted in humans under clinical conditions, the causality of the relationships between flavonoid intake and these health impacts as well as their exact mechanisms is still to be established.
